# Association between Pro‐oxidant‐Antioxidant balance and high‐sensitivity C‐reactive protein in type 2 diabetes mellitus: A Study on Postmenopausal Women

**DOI:** 10.1002/edm2.400

**Published:** 2022-12-28

**Authors:** Hassan Ehteram, Sara Raji, Mina Rahmati, Hanieh Teymoori, Samaneh Safarpour, Nahid Poursharifi, Mona Hashem Zadeh, Reza Pakzad, Hossein Habibi, Naser Mobarra

**Affiliations:** ^1^ Department of Pathology, School of Medicine Kashan University of Medical Sciences Kashan Iran; ^2^ Student Research Committee, Faculty of Medicine Mashhad University of Medical Sciences Mashhad Iran; ^3^ Metabolic Disorders Research Center, Department of Biochemistry Golestan University of Medical Sciences Gorgan Iran; ^4^ Department of Laboratory Sciences, School of Paramedical Sciences Mashhad University of Medical Sciences Mashhad Iran; ^5^ Department of Epidemiology, Faculty of Health Ilam University of Medical Sciences Ilam Iran; ^6^ Student Research Committee Ilam University of Medical Sciences Ilam Iran; ^7^ Department of Medical Laboratory Sciences Varastegan Institute for Medical Sciences Mashhad Iran; ^8^ Department of Clinical Biochemistry, School of Medicine Mashhad University of Medical Sciences Mashhad Iran

**Keywords:** diabetes, high‐sensitivity C‐reactive protein, post menopause, Pro‐oxidant‐antioxidant balance, vitamin D

## Abstract

**Introduction:**

Oxidative stress known as a predictive marker for cardiovascular and metabolic diseases could be measured through pro‐oxidant antioxidant balance (PAB). The present study aimed to evaluate PAB and its association with high‐sensitivity C‐reactive protein (hs‐CRP) in the serum of postmenopausal women with diabetes mellitus.

**Methods:**

In this case–control study, 99 diabetic and 100 healthy postmenopausal women without diabetes mellitus were recruited. Serum PAB values, hs‐CRP, lipid profile, insulin, and vitamin D levels were measured. Moreover, insulin resistance (HOMA‐IR, HOMA‐β and QUICKI), waist circumference (WC), waist‐to‐hip ratio (WHR), waist‐to‐height ratio (WHtR), and body mass index (BMI) were calculated.

**Results:**

Serum PAB, hs‐CRP, insulin resistance, HOMA‐β, QUICKI, low‐density lipoprotein cholesterol (LDL‐C), high‐density lipoprotein cholesterol (HDL‐C), and triglycerides (TG) levels were significantly higher in the postmenopausal women with diabetes mellitus, while there was no significant difference in the total cholesterol (TC), serum insulin, WC, WHR, WHtR and vitamin D levels between the groups. Pearson correlation coefficient showed that HDL‐C and insulin levels were directly correlated with serum PAB. Also, there was a significant direct relationship between LDL‐C and insulin levels and hs‐CRP. There was no meaningful relationship between serum insulin and vitamin D levels and other assessed parameters. Backward logistic regression showed a positive relationship between diabetes mellitus and serum PAB and an inverse relationship with serum HDL levels.

**Conclusions:**

Serum PAB, hs‐CRP concentration, and lipid profile were significantly different between postmenopausal women with and without diabetes mellitus. These differences may contribute to the development of coronary complications.

## Graphical abstract

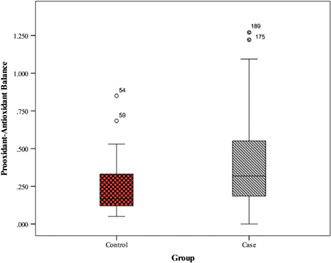

## 1 INTRODUCTION

An imbalance between the production of oxidants and their scavengers leads to oxidative stress (OS). OS may also stimulate the production of inflammatory factors, such as high‐sensitivity C‐reactive protein (hs‐CRP). Hs‐CRP is an inflammatory marker induced via cytokines, especially interleukin‐6 (IL‐6). OS and hs‐CRP are predictive markers of cardiovascular disease (CVD) and metabolic diseases, including type II diabetes.^1^, ^2^, ^3^

Type II diabetes mellitus (T2DM) is a severe, multifactorial and metabolic disease, which affects women more than men in many countries. T2DM increases CVD risk by 2–3 folds, which leads to a higher mortality rate than in non‐diabetic people.^4^ Moreover, OS increases in menopausal women, which is associated with loss of ovarian follicular function and oestrogen (E_2_) production because E_2_ has antioxidant activity.^5^ After menopause, the production of antioxidants is reduced, and OS increases.^6^, ^7^ Thus, menopause may be a risk factor for OS, CVD, osteoporosis, and diabetes. Although OS and inflammation are well established in postmenopausal women, there are limited studies about pro‐oxidant‐antioxidant balance (PAB), CRP levels and their association with insulin resistance in diabetic postmenopausal women.^1^, ^8^, ^9^

We sought to assess the serum PAB values using a modified PAB assay to measure the pro‐oxidant burden and antioxidant capacity. This study also evaluated hs‐CRP and whether serum PAB values are associated with hs‐CRP in diabetic postmenopausal women.

## 2 MATERIALS AND METHODS

### 2.1 Study groups

This case–control study was carried out on 99 postmenopausal women who had recently been diagnosed with only diabetes type II and attended the Women's Health Research Center in Gorgan, Iran. The control group included 100 healthy participants age‐matched to the patient group recruited between January 2017 and June 2018 for routine check‐ups. This group consisted of postmenopausal women with no diabetes. Clinical history and other relevant data were collected from all participants. They were excluded if they had taken vitamin supplements, hormones, anti‐inflammatory drugs, and fish oil capsules. Moreover, smokers and pregnant subjects were excluded from the study. Those suffering a myocardial infarction (MI), acute infection or any acute illnesses were excluded. One hundred and 99 subjects met the inclusion/exclusion criteria. They were informed about the study protocol, written consent was obtained from each participant and the research was approved by the Mashhad University of Medical Sciences Ethics Committee (NO: IR.MUMS.REC.1399.533).

### 2.2 Anthropometric parameters and blood collection

After overnight fasting, 5 ml of venous blood was drawn into EDTA and plain tubes, centrifuged at 2500 rpm for 15 min at room temperature, and serum was allocated to several microtubes and stored at −70°C until analysis. Furthermore, body weight, height, waist circumference (WC), and hip circumference (HC) were measured to calculate the waist‐to‐hip ratio (WHR), waist‐to‐height (WHtR), and body mass index (BMI) (kg/m^2^).

### 2.3 Biochemical analysis processing

Fasting glucose and lipid profile indices, including total cholesterol (TC), triglyceride (TG), and HDL‐C, were measured by enzymatic methods and commercial kits using the BT‐3000 Auto‐analyser (Biotechnica). Moreover, LDL‐C was indirectly evaluated in participants with the Friedewald formula.

The levels of insulin were assessed using commercial kits using a radioimmunoassay from the Immuno Nuclear Corporation (Stillwater). Insulin resistance was calculated using the HOMA equation: HOMA‐IR = [Fasting insulin (μIU/ml) fasting glucose (mM/L)]/22.5.

Also, homeostasis model assessment of β‐cell function (HOMA‐β) and quantitative insulin sensitivity check index (QUICKI) were used to assess β‐cell function and insulin sensitivity, respectively, as follows: HOMA‐β: (fasting plasma insulin [μU/ml] * 20)/(fasting blood glucose [mmol/l] – 3.5) and QUICKI: 1/(log fasting blood glucose [mmol/l] + log fasting plasma insulin [μU/ml]).

Furthermore, serum 25‐hydroxyvitamin D [25(OH) D] levels were assessed using a commercial ELISA kit (25‐Hydroxyvitamin D ELISA kit; Immuno Diagnostic Systems).

### 2.4 Measurements of hs‐CRP

The PEG (polyethylene glycol)‐enhanced immuno‐turbidometry method and commercially available kits on an Alcyon® analyser (Abbott) were used to measure hs‐CRP levels.

### 2.5 Assessment of PAB

Serum PAB values were measured in all subjects as previously described by Alamdari et al.^10^ In the first step, we added horseradish peroxidase enzyme and chloramine‐T as oxidizing agents to TMB. Redox index resulted in the combined activity of a colour cation (by oxidants) or reduced to a colourless compound (by antioxidants). In standard solutions, various proportions (0%–100%) of 250 μM hydrogen peroxide (as an oxidizing substance) were mixed with 3 mM uric acid (in 10 mM NaOH) (as an antioxidant). The absorption of 10 μl samples was measured with an enzyme‐linked immunosorbent assay (ELISA) reader at 450 nm for the reference, 630 nm, and the values of PAB were expressed in arbitrary (Hamidi. Koliakos [H.K]) units).

### 2.6 Statistical methods

The normality of the data was assessed by the Kolmogorov–Smirnov test. The mean and SD (for normal distribution) and median and interquartile range (IQR) (for non‐normal distribution) were used to describe the study variables. The independent student *t*‐test (for variable normality distribution) was used to compare the mean of study variables between case and control groups. A logistic regression method was used to determine the variables related to diabetes, including age, BMI, PAB, systolic blood pressure (SYSp), diastolic blood pressure (DIAp), GLUCOSE (Glc), insulin, InsulinR, TC, LDL, HDL, TG, hs‐CRP and vitamin D. Based on the Hosmer–Lemeshow method, simple logistic regression was utilized to determine the relationship between study variables and diabetes. Then, the variables with *p* < .2 were added to the final model and analysed using multiple logistic regressions. We used SPSS for Windows software (version 18 software package SPSS Inc). A *p*‐value less than .05 was considered statistically significant.

## 3 RESULTS

### 3.1 Participants' characteristics and demographic findings

All data showed a normal distribution. Demographic data, including age, BMI, SYSp and DIAp, were not significantly different between the two groups. Except for serum TC, insulin, vitamin D, WC, WHR and WHtR, other laboratory findings in diabetic subjects were significantly different from the non‐diabetic subjects (*p* < .05). Table 1 shows the features of the two groups.

**TABLE 1:** Demographic, cardiovascular risk parameters, and lipid profile indices in diabetic patients and healthy controls

Variables	Diabetic (*n* = 99)	Control (*n* = 100)	*p*‐value
Age (y)	65.33 ± 5.34	61.20 ± 5.990	.283
BMI (kg/m^2^)	26.6 ± 2.0	26.2 ± 2.4	.253
SYSp (mmHg)	13.03 ± 1.16	13.22 ± 1.20	.951
DIAp (mmHg)	7.49 ± 0.66	7.53 ± 0.75	.729
Glc (mmol/L)	198.72 ± 69.78	94.87 ± 5.68	<.001*
TC (mg/dl)	152.56 ± 10.77	150.19 ± 10.68	.122
LDL (mg/dl)	144.72 ± 33.27	131.70 ± 31.43	.005*
HDL (mg/dl)	46.03 ± 9.03	49.07 ± 10.10	.026*
TG (mg/dl)	166.19 ± 37.01	155.59 ± 26.76	.022*
Vit D (ng/ml)	19.29 ± 10.58	19.63 ± 8.12	.798
PAB (H.K)	0.40 ± 0.29	0.22 ± 0.13	<.001*
hs‐CRP (mg/dL)	5.11 ± 6.03	2.96 ± 3.07	.002*
Insulin R** (μU/mL × mmol/L)	4.10 ± 4.22	2.63 ± 2.52	.003*
HOMA‐ β (%)	1.09 ± 0.89	1.85 ± 1.38	<.001*
QUICKI	0.32 ± 0.03	0.36 ± 0.04	<.001*
Insulin (μU/ml)	9.33 ± 6.31	8.45 ± 6.48	.335
WC (cm)	95.49 ± 7.19	94.31 ± 10.3	.352
HC (cm)	102.54 ± 7.18	101.64 ± 5.88	.337
WHR	0.93 ± 0.07	0.93 ± 0.11	.767
WHtR	0.56 ± 0.04	0.55 ± 0.06	.717

*Note*: Values represent means ± SD. Comparisons were made by using Student's *t‐*test between groups.

Abbreviations: BMI: Body mass index; DIAp: Diastolic blood pressure; Glc: Glucose; HC: Hip Circumference; HDL‐C: High‐density lipoprotein cholesterol; HOMA‐β: Homeostasis Model Assessment‐β cell; hs‐CRP: High‐sensitivity C‐reactive protein; Insulin R: Insulin Resistance; LDL‐C: Low‐density lipoprotein cholesterol; PAB: Pro‐oxidant‐antioxidant balance; QUICKI: Quantitative Insulin Sensitivity Check Index; SYSp: Systolic blood pressure; TC: Total cholesterol; TG: Triglycerides; Vit D: Vitamin D; WC: Waist circumference; WHR: Waist‐to‐hip ratio; WHtR: Waist‐to‐height ratio.

*Significance was defined as *p* < .05. ** Insulin resistance was calculated using the HOMA equation (HOMA‐IR).

### 3.2 PAB values, hs‐CRP concentration and insulin resistance among postmenopausal women

Serum PAB levels in the diabetic subjects were significantly higher than in the control group (*p <* .001) (Table 1). Also, serum hs‐CRP concentrations were statistically different in the two groups (*p* = .002) (Table 1). Unsurprisingly, in diabetic women, there was a statistically significant difference in insulin resistance, HOMA‐β and QUICKI compared to non‐diabetic women (all *p <* .05), whereas no considerable difference was demonstrated between diabetic patients and healthy participants in serum insulin concentrations (*p* = .335).

### 3.3 The relationship between serum PAB values, BMI, and hs‐CRP concentrations and other laboratory parameters

**FIGURE 1:**
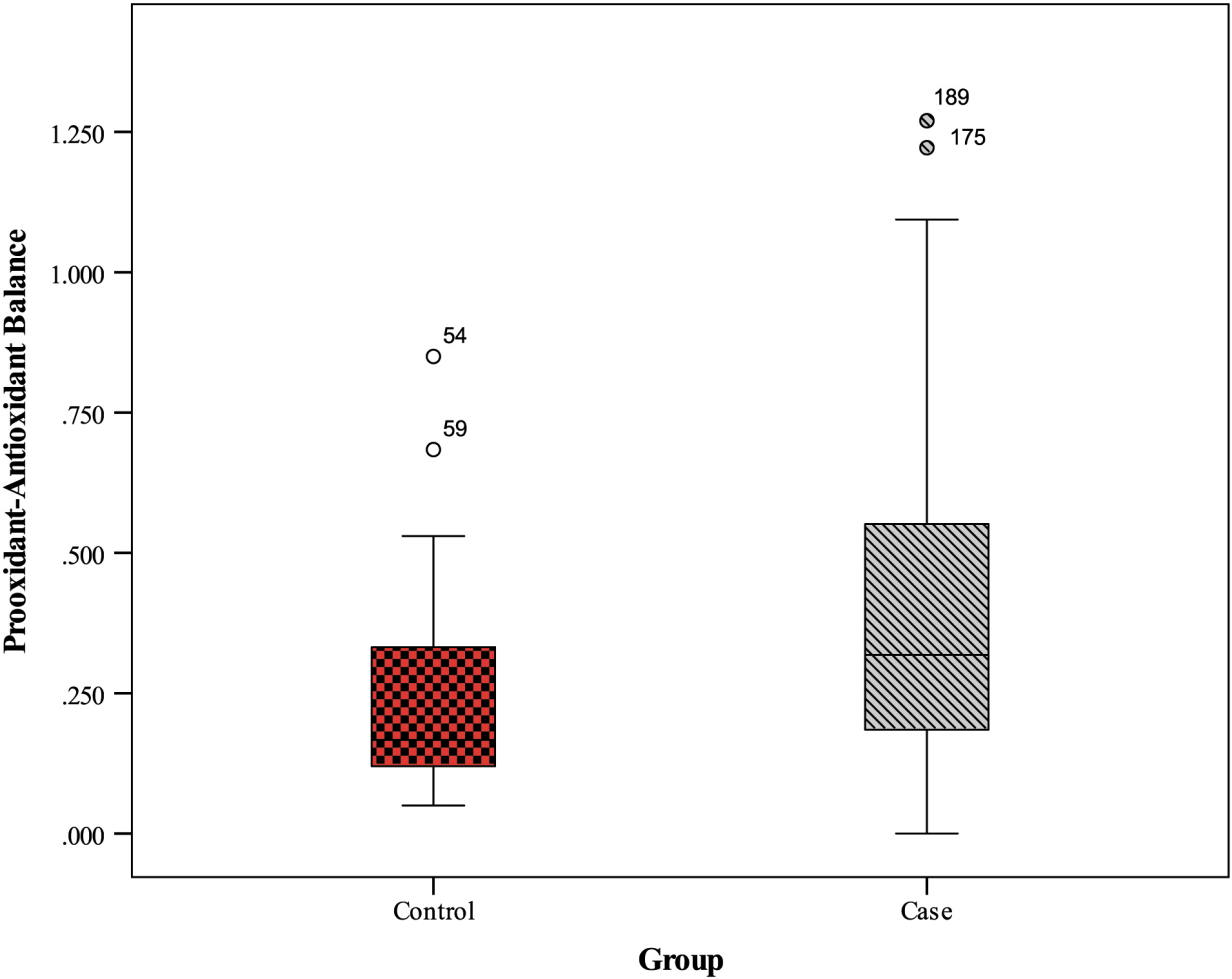
Pro‐oxidant‐antioxidant balance in case and control groups

**FIGURE 2:**
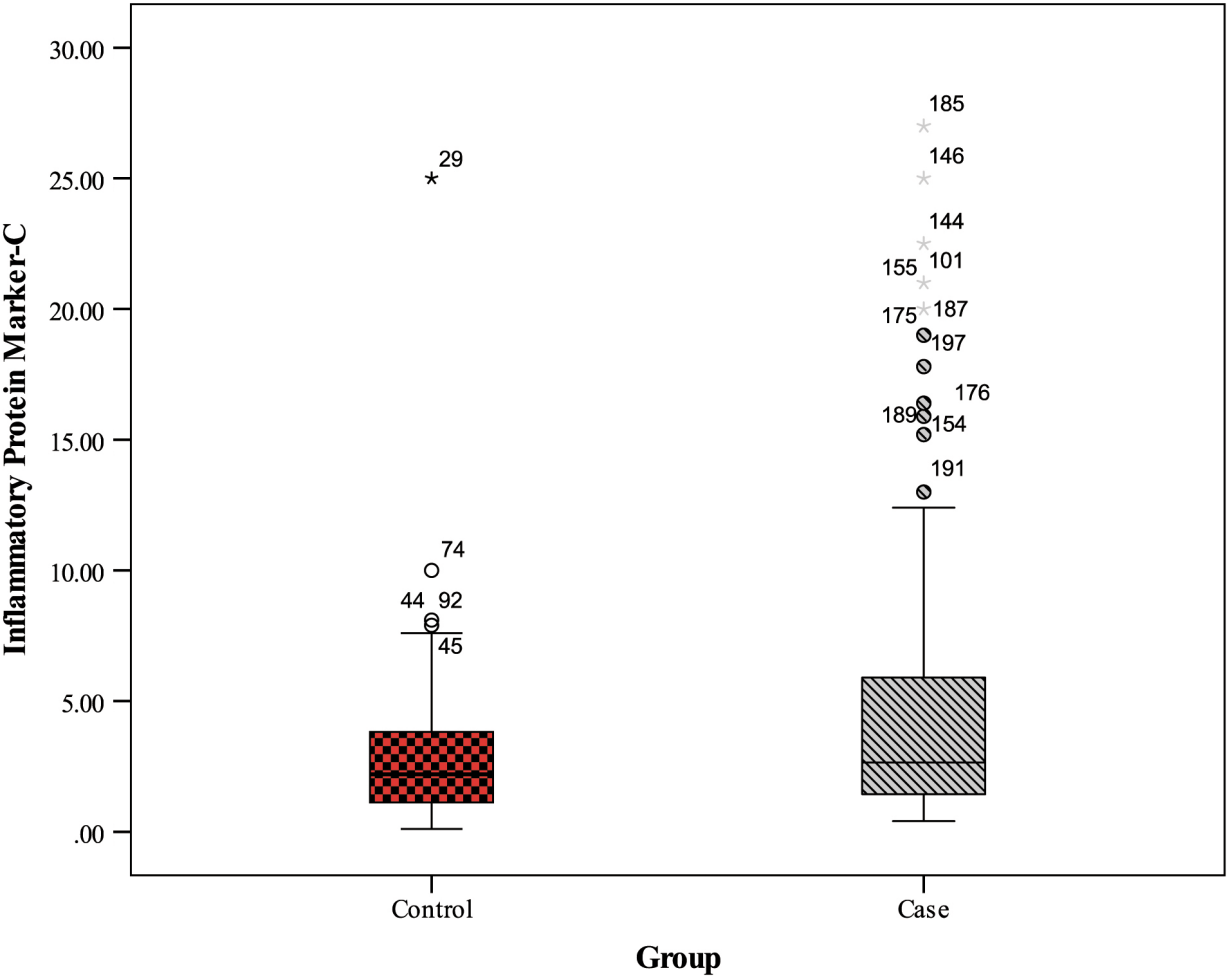
High‐sensitivity C‐reactive protein in patients and healthy subjects

As shown in Table 2, the Pearson correlation coefficient analysis was performed to evaluate the correlation between serum PAB values, BMI, hs‐CRP concentrations and other laboratory parameters. Scatter plots graphically showed a strong and positive uncorrected association between serum PAB values and hs‐CRP levels (*r* = .258 and *p* = .010) (Figure 1). We did not find any significant correlation between PAB values and insulin resistance (*r* = .095 and *p* = .347) (Figure 2). Moreover, serum PAB and hs‐CRP levels were positively correlated with serum insulin (*r* = .212, *p* = .035; *r* = .211, *p* = .037), respectively. Among the other study factors, a significant association was observed between serum PAB values and LDL‐C levels (*r* = .209, *p* = .038) and a negative correlation with HDL‐C levels (*r* = −0.224 and *p* = .026). Moreover, a comparison of the relationship between BMI and other values showed a significant correlation between BMI and TG levels (*r* = .207 and *p* = .042). In addition, we did not find any association between vitamin D levels and other laboratory parameters listed in this study.

**TABLE 2:** Pearson Correlation Coefficient between study variables in the case group

	Age	BMI	Insulin	InsulinR	LDL	HDL	TG	VitD	hs‐CRP	PAB
BMI	0.066	1								
	0.514									
Insulin	0.004	0.001	1							
	0.967	0.999								
InsulinR	0.022	−0.02	0.176	1						
	0.825	0.847	0.082							
LDL‐C	0.128	0.114	0.217*	−0.021	1					
	0.206	0.261	0.031	0.834						
HDL‐C	0.083	0.146	−0.055	0.054	0.039	1				
	0.415	0.148	0.59	0.598	0.703					
TG	0.039	0.207*	0.206*	−0.003	0.02	0.022	1			
	0.701	0.042	0.041	0.975	0.847	0.825				
VitD	0.073	0.004	−0.134	0.023	−0.131	−0.083	0.04	1		
	0.472	0.972	0.185	0.821	0.195	0.412	0.693			
hs‐CRP	−0.02	−0.143	0.211*	0.051	0.076	−0.026	0.025	0.005	1	
	0.848	0.159	0.037	0.618	0.457	0.801	0.805	0.963		
PAB	0.072	−0.023	0.212*	0.095	0.209*	0.224*	0.019	0.002	0.258*	1
	0.478	0.819	0.035	0.347	0.038	0.026	0.920	0.984	0.010	
Glc	0.055	0.105	−0.117	−0.138	0.136	−0.016	0.017	0.001	−0.063	0.034
	0.592	0.302	0.25	0.174	0.181	0.876	0.865	0.997	0.536	0.741

*Note*: Pearson correlation univariate analysis was used to test the relationship between parameters.

Abbreviations: BMI, Body mass index; Glc, Glucose; HDL‐C, High‐density lipoprotein cholesterol; hs‐CRP, High‐sensitivity C‐reactive protein; Insulin R, Insulin Resistance; LDL‐C, Low‐density lipoprotein cholesterol; PAB, Pro‐oxidant‐antioxidant balance; TG, Triglycerides; Vit D, Vitamin D.

*Significance was defined as *p* < .05.

### 3.4 Multiple logistic regressions

Logistic regression in the backward approach explained that InsulinR (OR: 1.16, p: 0.012), cholesterol (OR: 1.033; p: 0.047) and LDL‐C (OR: 1.017; p: 0.002) levels, and PAB values (OR: 174.89; *p* < .001) had a positive association with diabetes mellitus in patients compared to non‐diabetic women (Table 3). Moreover, these results showed that diabetes had an inverse association with HDL‐C (OR: −0.932; *p* < .001).

**TABLE 3:** Association between study variables and diabetes using multiple logistic regressions

	Variable	OR	95% CI	*p*‐value
Multiple logistic regression (entered approach, pseudo *R* ^2^ = .396)	InsulinR	1.151	1.019–1.300	.024
	Cholesterol	1.032	0.998–1.066	.063
	LDL‐C	1.015	1.004–1.026	.007
	HDL‐C	0.935	0.900–0.973	.001
	TG	1.010	0.999–1.022	.073
	Vit D	0.997	0.962–1.033	.877
	PAB	140.451	16.426–1200.97	<.001
	hs‐CRP	1.064	0.971–1.166	.186
Multiple logistic regression (backward approach, pseudo *R* ^2^ = .373)	Insulin R	1.165	1.034–1.313	.012
	Cholesterol	1.033	1.01–1.067	.047
	LDL‐C	1.017	1.006–1.028	.002
	HDL‐C	−0.932	0.897–0.968	<.001
	PAB	174.893	21.563–1418.518	<.001

Abbreviations: CI, Confidence interval; HDL‐C, High‐density lipoprotein cholesterol; hs‐CRP, High‐sensitivity C‐reactive protein; Insulin R, Insulin Resistance; LDL‐C, Low‐density lipoprotein cholesterol; PAB, Pro‐oxidant‐antioxidant balance; TG, Triglycerides; Vit D, Vitamin D.

Significance was defined as *p* < .05.

## 4 DISCUSSION

To our knowledge, this is the first case–control study to report PAB values and investigate the relationship between hs‐CRP levels and PAB values in postmenopausal women with and without diabetes mellitus. The main finding of the present study was the serum PAB and hs‐CRP elevation in diabetic postmenopausal women compared to non‐diabetic cases.

This finding is in accordance with earlier studies demonstrating the presence of systemic inflammation in diabetes. The increased level of OS is significantly associated with metabolic parameters in diabetic patients.^11^, ^12^ OS can be induced by inflammation^4^, ^13^; for example, higher concentrations of interleukin‐6 are an important stimulant for the production of hs‐CRP^14^ and inflammation can induce the production of free radicals.^15^ The present study showed that serum hs‐CRP levels were positively associated with serum PAB values in diabetic women. Moreover, earlier reports support the presence of high OS and hs‐CRP levels in stroke, cardiovascular and beta‐thalassemia patients.^16^, ^17^ There is strong evidence of the correlation between inflammation and OS because both factors contribute to the pathogenesis of diabetes.^18^ Moreover, diabetic postmenopausal women also had higher levels of blood glucose and HOMA‐IR index. In correlation with previous studies, dysregulated lipid metabolism in diabetics has been reported, which could be attributed to increased lipolysis due to impaired insulin function in adipose tissue. In addition, the accumulation of free fatty acids in the liver leads to the high hepatic synthesis of TGs and results in hypertriglyceridemia.^11^, ^19^ In this study, as shown by Barrett‐Connor et al.,^20^ no relationship was observed in total cholesterol between diabetic and non‐diabetic subjects. We did not find any significant difference between serum hs‐CRP, glucose, TG, LDL‐C levels, and BMI. These results were inconsistent with those of Yang et al.^21^ The reason may be due to the menopause subjects and the changes in the oestrogen hormone and its function in the liver. Moreover, parallel to our report, earlier reports have suggested that OS plays a major role in developing insulin resistance.^22^, ^23^

Consistent with many studies,^23^, ^24^ we can suggest that diabetic women have significantly altered lipid profiles than healthy postmenopausal subjects. Contrary to our work, many studies have reported that increased BMI values were strongly associated with hs‐CRP and OS levels.^25^ We suggest that independent of BMI, OS may also be an essential determinant of hs‐CRP levels in diabetic people. Therefore, the link between OS and hs‐CRP levels may involve pathways unrelated to BMI. In line with the study by Goodarzi et al.,^7^ there was no significant difference in BMI between the two groups. Moreover, consistent with Zaman et al.*,* the patient and control groups were overweight but not obese.^26^

Overweight women are not necessarily diabetic, and diabetes mellitus is not the only reason for the BMI increase in overweight type 2 diabetics; other factors may be involved. In addition, in line with our study, many studies have shown that people with diabetes also have a low BMI, and some have a very low BMI.^26^, ^27^ On the contrary, unlike some studies,^28^ our study found that diabetes mellitus in our diabetic patients was not necessarily dependent on insulin. Therefore, it can be concluded that in people with type 2 diabetes, other factors may have a role in the incidence of diabetes. Hence, it can alter insulin levels in people who have diabetes without a statistically considerable difference from healthy subjects.

In contrast to previous literature,^29^, ^30^ our findings demonstrated a positive relationship between serum hs‐CRP and insulin levels because inflammatory markers decrease insulin secretion and signalling in peripheral tissues. Moreover, interleukin‐6 decreases insulin signalling in the liver.^31^ In the present study, we found an irreversible correlation between PAB values and HDL‐C levels in line with A. Cagnacci et al.^32^ because oxidants can be reduced by the antioxidant enzyme paraoxonase carried by HDL‐C lipoproteins.^33^, ^34^ Moreover, we found a significant relationship between TG levels and BMI. This finding demonstrated that high TG can cause obesity and ultimately increase BMI in diabetic postmenopausal women. Besides, in contrast to the Cardiovascular Health Study and research by Mendall et al.*,* surprisingly, no relationship was found between hs‐CRP levels and BMI in women. Due to this controversy with the prior investigation, we think that diabetes in postmenopausal women can cause these outcomes. Our finding was in agreement with that of Kahn et al.,^35^, ^36^ indicating that diabetic postmenopausal women were characterized by insulin resistance. Moreover, it has been noted that insulin has a significantly negative relationship with higher hs‐CRP levels and PAB values. However, in Table 3, PAB values showed a positive correlation with LDL‐C levels and an irreversible association with HDL‐C levels. Therefore, the evidence supporting these results is that HDL cholesterol is the major lipoprotein carrier of antioxidant enzymes, and LDL is the main factor correlated with oxidative markers.

Our study had a few limitations. The present work focused only on PAB values. However, several other factors can affect these biochemical parameters in OS, including sex hormones. Another limitation was the small sample size.

## 5 CONCLUSIONS

We found significantly higher PAB values in diabetic postmenopausal women. Moreover, we demonstrated that increased hs‐CRP concentrations are strongly associated with PAB values, a reliable **OS** marker. This finding was independent of BMI and insulin resistance in diabetic postmenopausal women. Measurement of PAB hs‐CRP levels and other biochemical parameters may be a valuable marker for OS and inflammation and a helpful diagnostic factor to prevent injury and develop coronary artery disease. Future studies with larger sample sizes on PAB values and hs‐CRP may lead to the more practical use of these two markers in clinical diagnosis and follow‐up of diseases and better the quality of life for patients.

## AUTHOR CONTRIBUTIONS

**Hassan Ehteram:** Conceptualization (supporting); writing – review and editing (equal). **Sara Raji:** Data curation (equal); writing – original draft (equal); writing – review and editing (equal). **Mina Rahmati:** Data curation (equal); writing – review and editing (equal). **Hanieh Teymoori:** Data curation (equal); writing – review and editing (equal). **Samaneh Safarpour:** Data curation (equal); writing – review and editing (equal). **Nahid Poursharifi:** Data curation (equal); writing – review and editing (equal). **Mona Hashem Zadeh:** Data curation (equal); writing – original draft (equal); writing – review and editing (equal). **Reza Pakzad:** Formal analysis (lead); writing – review and editing (equal). **Hossein Habibi:** Writing – review and editing (equal). **Naser Mobarra:** Conceptualization (lead); supervision (lead); writing – review and editing (equal).

## FUNDING INFORMATION

This study is funded by Mashhad University of Medical Sciences (Grant No: 981826)

## CONFLICT OF INTEREST

The authors declared no conflicts of interest.

## ETHICAL APPROVAL

The Ethics Committee of Mashhad University of Medical Sciences approved the study (IR.MUMS.REC.1399.533).

## Data Availability

The data that support the findings of this study are available on request from the corresponding author. The data are not publicly available due to privacy or ethical restrictions.
